# Rapid Mechanistic Evaluation and Parameter Estimation of Putative Inhibitors in a Single-Step Progress-Curve Analysis: The Case of Horse Butyrylcholinesterase

**DOI:** 10.3390/molecules22081248

**Published:** 2017-07-26

**Authors:** Jure Stojan

**Affiliations:** Institute of Biochemistry, Faculty of Medicine, University of Ljubljana, Vrazovtrg 2, 1000 Ljubljana, Slovenia; stojan@mf.uni-lj.si; Tel.: +386-1-543-7649

**Keywords:** butyrylcholinestersae, inhibitors and reactivators, enzymic mechanism

## Abstract

Highly efficient and rapid lead compound evaluation for estimation of inhibition parameters and type of inhibition is proposed. This is based on a single progress-curve measurement in the presence of each candidate compound, followed by the simultaneous analysis of all of these curves using the ENZO enzyme kinetics suite, which can be implemented as a web application. In the first step, all of the candidate ligands are tested as competitive inhibitors. Where the theoretical curves do not correspond to the experimental data, minimal additional measurements are added, with subsequent processing according to modified reaction mechanisms.

## 1. Introduction

Cholinesterases have been extensively investigated for more than 60 years [1,2,3]. Due to the role of the acetylcholinesterases (AChEs; EC 3.1.1.7) in the termination of transmission at cholinergic synapses, much more attention has been focused on these, rather than the butyrylcholinesterases (BChEs; EC 3.1.1.8). However, the BChEs have become increasingly important, particularly as the first-line defense against nerve agent poisons and related pesticides, and also as a target enzyme for alleviation of symptoms in the later stages of Alzheimer’s disease [4,5]. These characteristics have thus promoted the continuous search for new compounds that can modulate the activities of these enzymes of the cholinesterase family [6,7].

In recent years, new approaches have been developed for the search for lead compounds through the availability of huge databases of existing natural and synthetic organic molecules [7]. With rapidly increasing computational power, search algorithms have been developed that allow all compounds in such databases to be surveyed according to specific criteria. These might include the size and charge of the molecules, their solubility, their predicted crossing of the blood–brain barrier, among many further possibilities [8]. A second level of software has also been introduced for the further insilico prediction of interactions/binding of the previously reduced number of candidates [9]. The resulting hits can then be subjected to rational selection in terms of some 50 to 100 potentially active compounds, as at this stage these can no longer be sorted by automatic means. The subsequent step is to test these potential hits for their biological activities, initially using the purified target; i.e., the enzyme. It should be stressed that such procedures have indeed recently resulted in the identification of several highly specific ligands, which have also been further improved by targeted chemical modifications in the following steps [6,7,8,9].

Here, the focus is on effective and rapid invitro determination of the biological activities of selected compounds as potential inhibitors of the system under investigation. This process is based on a single progress-curve measurement in the presence of each candidate ligand, to yield the corresponding kinetic parameters, and also to indicate the mode of action. The best-known classical reversible anti-cholinesterase inhibitors were included in this study, along with others that have shown more complex interactions. For reliable verification of the mode of action of each of these compounds, the three-dimensional (3D) structures of most of them in complexes with at least one cholinesterase are available [10,11,12].

## 2. Results and Discussion

Hundreds of ligands have been tested as inhibitors of cholinesterases over recent decades, with their 3D structures in complexes with cholinesterases solved in many cases. Most of these studies were stimulated during searches for new nerve agents, and for compounds that might provide protection against them. Recently, several studies have concentrated on inhibitors with neuroprotective roles, and especially compounds that specifically interact with BChEs [7,13,14,15]. To reach this goal, all serious candidate ligands have to be tested in vitro on the target enzyme(s). Although new, more or less automated technologies equipped with expensive robots can furnish a huge amount of information in a relatively short period of time, all of this information must be processed, with the main aim being to single out only the potentially interesting compounds. While most screening tests use IC_50_ as a determining criterion, it is clear that this is a relatively limited system. Indeed, an IC_50_ can only provide reliable conclusions if the compound acts in a non-competitive fashion. Otherwise, the IC_50_ can be very misleading, unless the concentration and type of substrate are also given. In contrast, the dissociation constant is universal, and thus not substrate dependent. Even more importantly, when classical initial rate analysis is used, any slow event during the interaction between the ligand and the target protein might be overlooked. Therefore, by making use of multiple progress curve analysis, as suggested here, it is exactly these drawbacks that are avoided. The selected inhibitors in this study are well-known compounds, and most of them come with solved 3D structures in complexes with cholinesterases, for which the kinetics parameters and reaction mechanisms have been reported.

**Figure 1:**
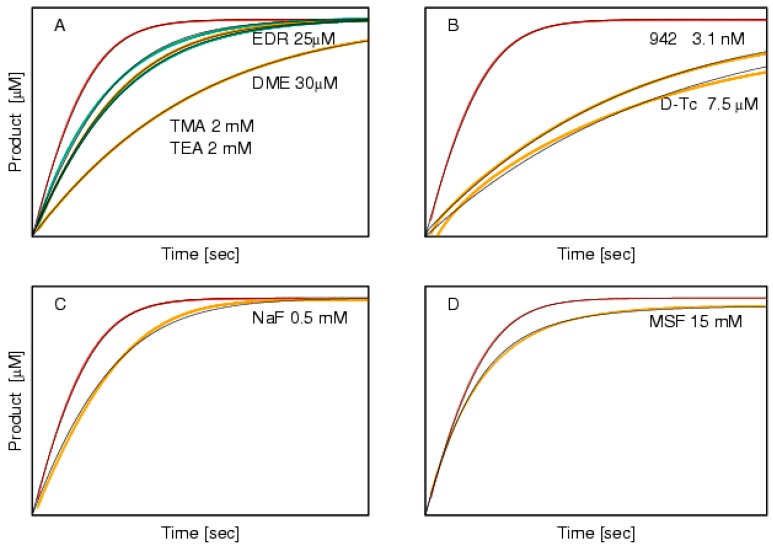
Progress curves (to 600 s) for the hydrolysis of 50 μM BSCh in the absence (red curves, in all panels) and presence of the eight inhibitors. (**A**) The reversible competitive inhibitors tetramethylammonium (TMA, upper green), tetraethylammonium (TEA, lower green) edrophonium (EDR, upper orange), and decamethonium (DME, lower orange); (**B**) The slow binder *d*-tubocurarine (D-Tc, lower orange) and the tight binder 942 (upper orange); (**C**) The mixed reversible inhibitor sodium fluoride (NaF); (**D**) The irreversible inhibitor methanesulfonylfluoride (MSF).

Figure 1 shows the progress curves for each of the eight inhibitors investigated here, in the presence of the 50 μM butyrylthiocholine iodide (BSCh) starting concentration. Within this substrate concentration, cholinesterases follow simple hyperbolic kinetics and the evolving products (thiocholine and butyrate) do not affect the hydrolysis. Figure 1 is divided into four panels for clarity, and also on the basis of the thorough inspection of their shape and the analysis using the ENZO application, according to competitive inhibition.

The theoretical curves in Figure 1A support the experimental data, which indicate that tetramethylammonium iodide (TMA), tetraethylammonium iodide (TEA), edrophonium chloride (EDR) and decamethonium bromide (DME) indeed act as competitive inhibitors of BSCh hydrolysis by horse BChE. This is in line with the (expected) positioning of EDR and DME in the active site of horse BChE, although the structures were actually solved for *Torpedo californica* AChE. A similar reasoning leads to the conclusion that the positive charged TMA and TEA are accommodated above E197 of horse BChE, where they form additional cation-Π interactions with W82 in the same way as seen for BSCh (Protein Data Bank code: 1P0P, [12]). It should be mentioned here, that in the presence of higher substrate concentrations any ternary or quaternary complexes between acylated enzyme, the inhibitor, and the substrate are expected. Consequently, uncompetitive or parabolic component in the inhibition pattern can be introduced, revealing substrate inhibition/activation but also paradoxical activation by an inhibitor [13].

The two theoretical curves in Figure 1B, however, do not follow the experimental data. Here, it can be seen that the initial portions of the curves in particular deviate substantially from the data. As *d*-tubocurarine was previously defined as a slow binding inhibitor of horse BChE, its action was interpreted as instantaneous binding followed by a slow isomerization step [14]. Therefore, such an isomerization step was added to the final analysis, and then the theoretical curve followed the experimental data (run the evaluation at http://enzo.cmm.ki.si/kinetic.php?uwd=170623269&load=true). On the other hand, the reason for the similar shape of the progress curve in the presence of 942 is different. This is a tight binding inhibitor that acts at concentrations close to that of BChE. Consequently, a very high affinity that is characterized by a low dissociation rate constant (*k_off_*, 0.027 s^−1^) causes the slow onset of inhibition at very low concentrations despite a relatively high association rate constant (*k_on_*, 6.2 × 10^7^ M^−1^ s^−1^).

**Figure 2:**
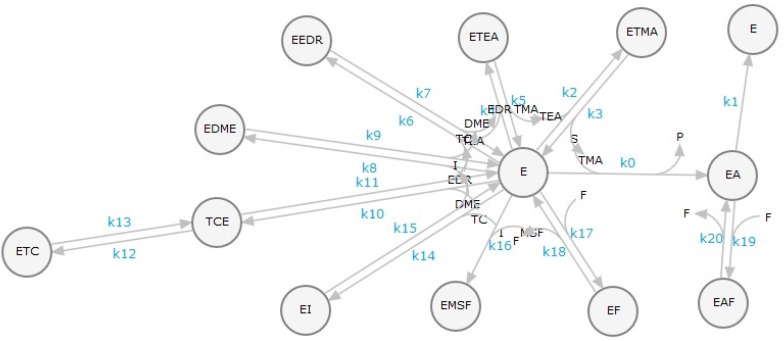
Reaction scheme for the simultaneous analysis of four competitive reversible enzyme inhibitors: a mixed reversible enzyme inhibitor, an irreversible inhibitor, a slow binding inhibitor, and a tight binding reversible inhibitor in the presence of the substrate. In this scheme, *E* is horse BChE, the reversible inhibitors are tetramethylammonium(TMA), tetraethylammonium (TEA), edrophonium (EDR), and decamethonium (DME), the mixed inhibitor is sodium fluoride (F), the slow binding inhibitor is *d*-tubocurarine (TC), which acts in two steps, as an instantaneous step followed by a slow isomerization, the irreversible inhibitor is methanesulfonylfluoride (MSF), and the tight binderis 942 (I). *S* represents the substrate BSCh and *P* represents thiocholine, a detection product. k_0_–k_20_ are second and first order rate constants. The scheme was generated by the web application ENZO at http://enzo.cmm.ki.si/kinetic.php?uwd=170623269&load=true.

The action of the small irreversible inhibitor, methanesulfonylfluoride (MSF), is shown in Figure 1D, and this was very easy to diagnose: the plateau of the curve is lower than the plateau of the curve in the absence of MSF, because the concentration of MSF used inactivates horse BChE before the total added substrate can be hydrolyzed. This is why the step for MSF in the reaction scheme (Figure 2, *k_16_*) is modelled as an irreversible one. It can also be stressed here that the initial rate in this curve that was obtained at a relatively high concentration (15 mM MSF) would not recognize MSF as an inhibitor, although MSF has successfully passed stage 2 of clinical trials for approval as an anti-Alzheimer’s disease drug [16].

### SodiumFluoride as an Inhibitor of Butyrylthiocholine Iodide Hydrolysis by Horse Butyrylcholinesterase

**Figure 3:**
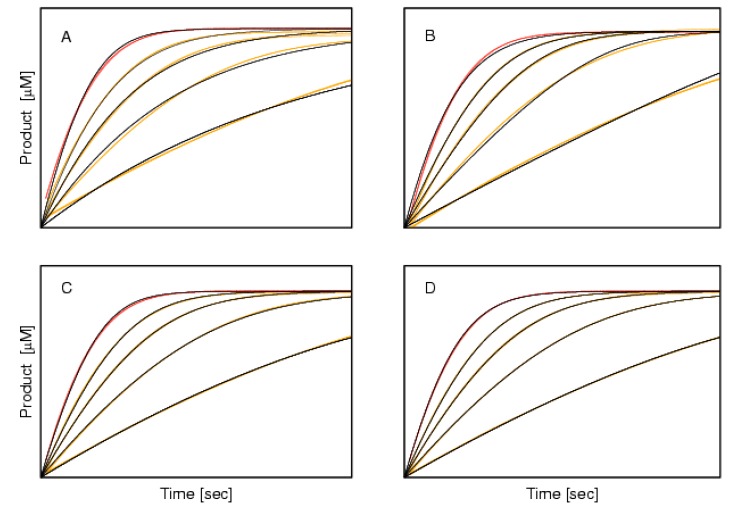
Progress curves for the inhibition of 50 μMBSCh hydrolysis by horse BChE in the absence (red) and presence (orange) of sodium fluoride (0.25, 0.5, 1 and 2.5 mM, from the highest to the lowest, respectively). The black curves are theoretical, obtained for four types of reversible inhibition. (**A**) Competitive; (**B**) Uncompetitive; (**C**) Non-competitive; (**D**) Mixed. Note the agreement between the experimental and fitted curves.

The fluoride anion is a well-known BChE inhibitor [17,18]. The solved 3D crystal structure of the fluoride anion in complex with human BChE showed that it is positioned in the oxyanion hole of the BChE active site (PDB code: 2XMC, [19]), adjacent to the hydroxyl group of the catalytic serine. This position thus prevents the carbonyl oxygen of the substrate from positioning appropriately for hydrolysis. As a consequence, it would be reasonable to expect competitive inhibition for sodium fluoride. However, the progress curve for the hydrolysis of BSCh by horse BChE in the presence of 0.5 mM sodium fluoride does not support this conclusion (Figure 1C). It can be seen that the initial part of the theoretical curve lies above the experimental curve, and that the approach to the plateau is slower than the approach of the experimental curve. Such a course indicates that the inhibition becomes weaker as the substrate concentration decreases, which clearly contradicts competitive inhibition. To provide an explanation for this, measurements in the presence of three additional sodium fluoride concentrations were conducted under otherwise identical conditions. Then, the progress curves obtained in the absence and presence of these four sodium fluoride concentrations were analyzedaccording to each of four classical reversible inhibition mechanisms. The results are shown in Figure 3, and these are in line with the initial findings. Indeed, the best agreement between the experimental and theoretical curves was achieved when the data were modelled to the mixed inhibition, which yielded an uncompetitive component that was 2.4-fold stronger than the competitive component (Table 1). Taking into account this finding and the solved 3D structure of fluoride complexed with BChE, it appears that the positively charged substrate, BSCh, serves as a vehicle for the delivery of the otherwise repelled negatively charged particles (i.e., fluoride) into the active site of the cholinesterases. Moreover, the electrostatic attraction between the positive moiety of BSCh and other halogen anions besides fluoride is much weaker, so their concomitant entrance into the active site is very unlikely. Once the fluoride anion is delivered to the bottom of the active site, it is substituted by the E197 side chain and the released fluoride can easily enter the adjacent oxyanion hole (compare [18]). The outstanding binding characteristics of the fluoride anion were compared with analogous measurements with sodium iodide, which showed no inhibition at 0.5 mM sodium iodide (data not shown). On the other hand, it might be argued that the iodide ion was already present in these measurements, because BSCh iodide was used as substrate. The absence of inhibition with the 10-fold higher iodide concentration suggests that the similar ‘co-transport’ of iodide into the active site does not occur. This appears to be because, in contrast to fluoride, iodide is largely dissociated from the positively charged moiety of the substrate BSCh in the bulk solute.

With this study, we have shown that simultaneous progress-curve analysis of a number of different inhibitors can provide basic information on their binding properties, even with a single kinetics measurement at hand. Not only can the inhibition constants be determined, but also the mode of action. In most cases, the findings can be interpreted through correlations with the available crystallographic information. When, however, the theoretical and experimental kinetics contradict, the rational explanation can still be sought through minimal additional experiments. Indeed, in the case of the cholinesterases, the large amount of different data produced previously helps in reaching an adequate interpretation. Nevertheless, the analysis itself is of paramount importance. In comparison to initial rate data, the complete progress curves include more mechanistic information by far [20], and with the appropriate analysis none of this kinetics information is wasted. However, it must be remembered, that a single progress curve measurement analysis might be insufficient if a peculiar pattern is encountered. Here, for instance, the used low substrate concentration essentially simplifies the actual happenings in the active site in comparison to the events going on at higher substrate concentrations [21,22]. Nevertheless, it seems that for fast screening of a long list of new putative inhibitors, the suggested method is the method of choice.

In this report, a web application designed exactly for such cases was used [23]. This is based on a user-friendly graphical interface for the easy drawing of the reaction mechanisms, which is linked to a numerical integration algorithm for the solving of systems of differential equations and for the corresponding parameter estimation. In an alternative analysis, the integrated Michaelis–Menten equation derived by Goličnik in 2013 can be used [24]. Although this equation can only handle classical reversible inhibitors of different types (see Appendix A), it does clearly reveal deviations, as shown for *d*-tubocurarine and 942 here.

## 3. Methods and Materials

### 3.1. Chemicals

Purified horse BChE was from Worthington Biochemical Corporation, dithio-bis-di-nitro benzoic acid and butyrylthiocholine iodide (BSCh) were from Sigma, tetramethylammonium iodide (TMA), tetraethylammonium iodide (TEA), edrophonium chloride (EDR), and methanesulfonylfluoride (MSF) were from BDH; decamethonium bromide (DME) was from Koch Light Laboratories; sodium fluoride and *d*-Tubocurarine were from Fluka AG. *N*-((1-benzylpiperidin-3-yl)methyl)-*N*-methylnaphthalene-2-sulfonamide (942) was synthesized in the Faculty of Pharmacy, University of Ljubljana (Ljubljana, Slovenia). All other reagents used were of analytical grade. The experiments were carried out at 25 °C in 25 mM phosphate buffer, pH 7.0.

### 3.2. Inhibition of Butyrylthiocholine Iodide Hydrolysis by Horse Butyrylcholinesterase

A series of compounds were tested by measuring the progress curves for BSCh hydrolysis by horse BChE in the absence and presence of each compound at a single concentration over 10 min, in a 0.6 mL cuvette, using the method of Ellman et al. [25]. These included the four classical inhibitors of 2 mM TMA, 2 mM TEA, 25 μM EDR, and 30 μM DME, and also 0.5 mM sodium fluoride, 7.5 mM *d*-tubocurarine (slow binder), 3.13 mM 942 (tight binder), and 15 mM MSF (irreversible inhibitor). The concentration of horse BChE, which was always added last, was ~1 nM, for hydrolysis of 50 μM BSCh to completion (in most cases), in the presence of 1 mM dithio-bis-di-nitro benzoic acid. Reaching the plateau was important to get an exact estimate of the actual initial BSCh concentration. This was achieved by conversion of the change in absorbance at 412 nm of the resulting thio-nitro benzoate, using a molar absorption coefficient of 13,800 mol^−1^ cm^−1^. All of the measurements were conducted using a conventional spectrophotometer (Lambda 45/Vis; Perkin-Elmer), and a 10-s lag period was added before the start of each measurement. The progress curves were then extrapolated to start at the origin prior to the analysis.

### 3.3. Theoretical Basis of the Kinetics Analysis

The progress-curve analysis was performed using the ENZO web application (Figure 2), implemented at www.enzo.cmm.ki.si [23]. For rapid determination of the kinetics mechanisms and parameters, the Van Slyke–Cullen reaction scheme [26] for substrate hydrolysis by horse BChE was combined with a reversible competitive inhibition mechanism, as expected for active-site-directed ligands. For MSF, a well-known irreversible inhibitor, a simple irreversible second-order reaction was used. In the initial evaluation, all of the second-order binding rate constants (*k_on_*’s), although not the constant for MSF, were set to the diffusion rate-limited value of 2 ×10^8^ M^−1^ s^−1^. Therefore, only the first-order dissociation rate constants, together with the substrate specificity constant *k_cat_*/*K_m_*, the initial substrate concentration, and the added horse BChE active-site concentration, had to be evaluated. The *k_cat_* of 663 s^−1^ for the hydrolysis of BSCh by horse BChE was taken from a previous study performed under the same conditions [27].

### 3.4. Sodium Fluoride Inhibition of Butyrylthiocholine Iodide Hydrolysis by Horse Butyrylcholinesterase

The experimental time-course of sodium fluoride inhibition of BSCh hydrolysis by horse BChE clearly deviated from reversible competitive inhibition. Therefore, a similar experiment was performed, but in the presence of three additional sodium fluoride concentrations (0.25, 1, 2.5 mM). Each of these measurements was performed twice in a row to determine the reliability of the data obtained. The progress curve analysis was then performed using four types of classical inhibition mechanisms: competitive, uncompetitive, non-competitive, and mixed. Again, a 10-s lag period was added to the time-course of each measurement prior to the analysis using the ENZO web application.

## 4. Conclusions

For rapid kinetics evaluation of a number of putative inhibitors, a single progress-curve measurement for each candidate is suggested. A set of curves in the presence of different compounds is analyzed in a simultaneous evaluation run, to estimate the inhibition parameters and define the inhibitors with unconventional mechanistic patterns. These latter can be further processed, in some cases with just a few additional experiments. A subsequent repetition of the measurements under identical conditions, like the 10 curves shown in Figure 3, support the reliability of the information obtained.

## Figures and Tables

**Table 1:** Kinetics parameters for the inhibition of horse BChE by the eight inhibitors ^a^.

Inhibitor	Kinetics Parameter					
	*K_i_*	*K_ii_*	*K_on_*	*K_off_*	*K_+isomerization_*	*K_-isomerization_*
Tetramethylammonium	2.4 mM					
Tetraethylammonium	1.5 mM					
Edrophonium (1ACK)	21 μM					
Decamethonium (1ACL)	7.6 μM					
Sodium fluoride * (2XMC)	0.83 mM	0.34 mM				
*d*-Tubocurarine	5.1 μM				0.029 s^−1^	0.010 s^−1^
942 (5DYT)	0.43 nM		6.2 M^−1^ s^−1^	0.003 s^−1^		
Methanesulfonylfluoride (5EHX)			0.39 s^−1^			

* from Figure 3. ^a^ :For standard errors, the application can be run at: http://enzo.cmm.ki.si/kinetic.php?uwd=170623269&load=true.

## Appendix A

An explicit list of reactions implemented in the Scheme as depicted in Figure 2 is:

E+S →EA+P→E
E+TMA ↔ETMA
E+TEA ↔ETEA
E+EDR↔EEDR
E+DME↔EDME
E+F↔EF
E+TC↔TCE↔ETC
E+MSF →EMSF
E+I↔EI

*E* is horse BChE, *TMA* stands fortetramethylammonium, *TEA* is tetraethylammonium, *EDR* is edrophonium, *DME* is decamethonium, *F* is sodium fluoride, *TC* is *d*-tubocurarine, which acts in two steps, as an instantaneous step followed by a slow isomerization, *MSF* is methanesulfonylfluoride and the *I* is 942. *S* represents the substrate BSCh and *P* represents thiocholine, a detection product.

In the suggested analysis the substrate hydrolysis (first reaction) was common to all tested inhibitors and so the two determined parameters (*k_cat_*/*K_m_* and *k_cat_*).

An alternative analysis of the curves in the presence of classical reversible inhibitors uses the integrated Michaelis–Menten equation as derived by Goličnik [22]:

$$P_{t}=\;S_{0}\;-\;K_{M}\times \left(1.4586887\times \ln\left(1.2\times \frac{S_{0}\times e^{\left(\frac{S_{0}-V_{max}*t}{K_{M}}\right)}}{K_{M}\ln\left(2.4\times \frac{S_{0}\times e^{\left(\frac{S_{0}-V_{max}*t}{K_{M}}\right)}}{K_{M}\ln\left(1+2.4*\frac{S_{0}\times e^{\left(\frac{S_{0}-V_{max}*t}{K_{M}}\right)}}{K_{M}}\right)}\right)}\right)-\;0.4586887\times \ln\left(2\times \frac{S_{0}\times e^{\left(\frac{S_{0}-V_{max}\times t}{K_{M}}\right)}}{K_{M}\ln\left(1+2\times \frac{S_{0}\times e^{\left(\frac{S_{0}-V_{max}\times t}{K_{M}}\right)}}{K_{M}}\right)}\right)\right)$$

In this equation, *K_M_* is substituted by *K_M_ ×* (1 *+* [*I*]*/K_i_*) to get the progress curve equation in the presence of a competitive inhibitor, or *V_max_* is substituted by *V_max_/*(1 *+* [*I*]*/K_i_*) for non-competitive inhibition. If *V_max_* is substituted by *V_max_/*(1 *+* [*I*]*/K_ii_*) and *K_m_* by (1 *+* [*I*]*/K_i_*)*/*(1 *+* [*I*]*/K_ii_*), the equation for mixed inhibition is obtained.
